# Relationship between high-sensitivity C-reactive protein/lymphocyte ratio and post-thrombolysis short-term outcome in acute ischemic stroke patients

**DOI:** 10.3389/fnagi.2026.1839899

**Published:** 2026-05-29

**Authors:** Ruixiu Zhu, Gang Chen, Yaya Fei, Jiajie Lu, Chenxue Xu, Yuying Xia, Zhiyu Zhang, Xindi Jiang, Shuaiyu Chen, Xiaohao Zhang, Yi Xie, Jianhua Song, Yuechun Wang, Yachi Gong, Pengyu Gong

**Affiliations:** 1 Department of Geriatrics, Nantong Third People’s Hospital, Affiliated Nantong Hospital 3 of Nantong University, Nantong, Jiangsu, China; 2 Department of Neurology, Haimen Hospital Affiliated to Nantong University, Nantong, Jiangsu, China; 3 Department of Neurology, Affiliated Hospital of Nantong University, Medical School of Nantong University, Nantong, Jiangsu, China; 4 Department of Neurology, Nanjing First Hospital, Nanjing Medical University, Nanjing, Jiangsu, China; 5 Department of Neurology, Nantong Third People’s Hospital, Affiliated Nantong Hospital 3 of Nantong University, Nantong, Jiangsu, China

**Keywords:** acute ischemic stroke, biomarker, high-sensitivity C-reactive protein/lymphocyte ratio, intravenous thrombolysis, short-term functional outcome

## Abstract

**Objectives:**

High-sensitivity C-reactive protein / lymphocyte ratio (hsCLR) was emerged as a promising novel biomarker for various diseases. However, the relationship between hsCLR and short-term outcome in acute ischemic stroke (AIS) patients treated with intravenous thrombolysis (IVT) remained unclear.

**Methods:**

This multicenter study included 904 AIS patients undergoing IVT. Multivariate logistic regression and restricted cubic spline (RCS) analysis were used to explore the association between hsCLR levels and unfavorable short-term outcomes after thrombolysis. Receiver operating characteristic (ROC) analysis was performed to evaluate the prognostic performance of hsCLR. Net reclassification improvement (NRI) and integrated discrimination improvement (IDI) analyses were further conducted to assess the incremental prognostic value of adding hsCLR to conventional risk models.

**Results:**

We found that patients with higher levels of lnhsCLR were more likely to experience short-term unfavorable outcome (*P* < 0.001). The multivariate logistic model revealed that increased levels of lnhsCLR [odds ratio (OR): 2.33, 95% CI (Confidence interval): 1.42–3.81, *P* < 0.001] were significant associated with short-term unfavorable outcome (hsCLR quartile 4 vs. quartile 1) after the adjustment of confounding factors. RCS analyses exhibited a linear pattern (*P* for overall = 0.002; *P* for nonlinear = 0.391). Moreover, NRI and IDI analyses demonstrated that adding lnhsCLR to the conventional risk model provided incremental prognostic value, with an NRI of 21.22% (*P* = 0.002) and an IDI of 1.21% (*P* = 0.002).

**Conclusion:**

Elevated baseline hsCLR is associated with unfavorable short-term outcomes and may serve as a promising prognostic biomarker in AIS patients undergoing intravenous thrombolysis.

## Introduction

Ischemic stroke (IS), the most common type of stroke, constitutes approximately 70% of all stroke cases (Campbell et al., 2019; GBD 2021 Stroke Risk Factor Collaborators, 2024). It is distinguished by its abrupt onset, high incidence, and significant disability, thus representing a major global health challenge associated with increased mortality rates and substantial disability. Prompt revascularization is critical for improving neurological functional outcomes. Intravenous thrombolysis (IVT) is considered the standard treatment when administered within 4.5 h of stroke onset, as it may be able to dissolve thrombi and restore cerebral blood flow (Powers et al., 2019). However, there is significant inter-individual variability in the efficacy of IVT. A proportion of patients fail to achieve successful recanalization or experience futile recanalization, resulting in unfavorable post-thrombolysis functional outcomes (D’Anna et al., 2025; Deng et al., 2022). Consequently, the identification of effective biomarkers is imperative to enhance prognostic accuracy and guide personalized interventions, thereby improving outcomes for patients with acute ischemic stroke (AIS) undergoing IVT.

Inflammation constitutes a critical pathological mechanism in cerebral ischemic injury, with inflammatory mediators playing an essential role in the initiation and progression of IS (Borlongan, 2023; Candelario-Jalil et al., 2022; Cicchitano et al., 2019; Gong et al., 2021; Ohashi et al., 2023; Simats and Liesz, 2022). An increasing body of evidence underscores the prognostic significance of various serum inflammatory markers in IS. C-reactive protein (CRP), an acute-phase protein produced by hepatocytes, functions as a sensitive marker of systemic nonspecific inflammation. Researches showed that elevated CRP levels might be significantly correlated with poor outcomes in AIS patients (den Hertog et al., 2009; Shantikumar et al., 2009; Yu et al., 2019). Additionally, diminished levels of lymphocyte counts are independently linked to neurological deterioration and unfavorable prognosis, suggesting compromised immune defense and repair mechanisms (Macrez et al., 2011; Wang et al., 2023). Nevertheless, reliance on a single biomarker may be vulnerable to interference from non-stroke-related factors such as infection or trauma, and may not adequately reflect the functional status of the immune-inflammatory system.

Composite inflammatory indices combining CRP and lymphocyte counts have shown prognostic value in AIS. Recent studies have demonstrated that the CRP / lymphocyte ratio (CLR) and CALLY index are associated with stroke severity and functional outcomes in patients with AIS (Demirel and Akunal Türel, 2023; Dogan et al., 2025; Lu et al., 2025; Pan et al., 2025; Zhu et al., 2025). However, the majority of prior studies used conventional CRP rather than high-sensitivity CRP (hsCRP), which provides greater sensitivity for low-grade inflammation in the acute stroke phase. Furthermore, the prognostic significance of hsCLR specifically in patients treated with IVT remained unclear. Therefore, this study aimed to prospectively investigate the association between baseline hsCLR levels and short-term functional outcome at discharge in AIS patients undergoing IVT.

## Materials and methods

### Study design

A total of 1,315 participants aged 18 years or older with AIS, who received IVT within 4.5 h were initially enrolled between June 2022 and November 2025 from three tertiary hospitals: the Affiliated Hospital of Nantong University, Nantong Third People’s Hospital, and Nanjing First Hospital. Exclusion criteria were as follows: (1) Severe inflammatory or infectious diseases within 2 weeks preceding stroke onset; (2) Severe liver and kidney dysfunction; (3) Incomplete data of hsCRP or lymphocyte. This was a multicenter, prospective, observational study. Data collectors across all centers underwent standardized training. Data collection followed consistent case inclusion/exclusion criteria, standardized recording protocols, and quality control measures. After applying the predefined inclusion and exclusion criteria, 411 patients were excluded, and 904 patients were ultimately included in the final statistical analysis.

**FIGURE 1:**
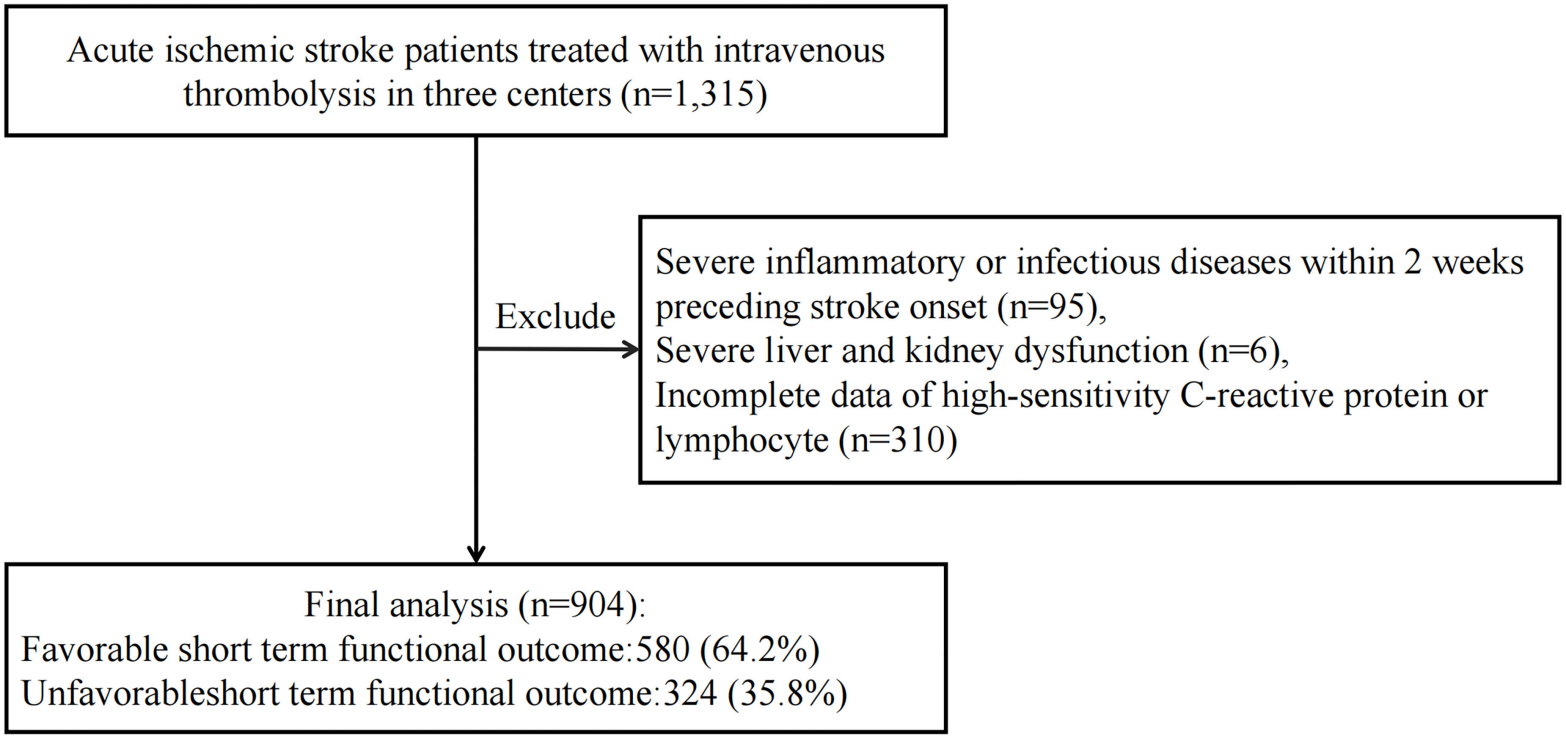
The flowchart of participants selection.

A total of 904 participants were ultimately included in the analysis. This study was conducted in compliance with the Declaration of Helsinki and approved by the Ethics Review Committee of Affiliated Hospital of Nantong University, Nantong Third People’s Hospital, and Nanjing First Hospital. The detailed screening process of the study participants is visually depicted in Figure 1.

### Data acquisition and outcome assessment

The study data included age ( ≥ 65 vs. <65), gender (female vs. male), current smoking (yes vs. no), current drinking alcohol (yes vs. no), history of diabetes mellitus (DM) (yes vs. no), history of hypertension (yes vs. no), history of atrial fibrillation (AF) (yes vs. no), history of coronary heart disease (CHD) (yes vs. no), proximal arterial occlusion (PAO) (yes vs. no), National Institutes of Health Stroke Scale (NIHSS) group (0–5 vs. ≥ 6), stroke subtype, onset to treatment time (OTT) (< 3 h vs. 3–4.5 h), medication use (antiplatelet, anticoagulation), hsCRP and lymphocyte count collected from the hospital information system. Stroke subtype was classified according to Trial of Org 10172 in Acute Stroke Treatment (TOAST) criteria (Adams et al., 1993). The hsCLR is calculated by dividing the hsCRP by lymphocyte count, both measured from blood samples: the levels of hsCRP (mg/l) / lymphocyte count (10^9^ /l). The outcome was the short-term unfavorable functional outcome, defined as an mRS score of 3–6 at discharge (Banks and Marotta, 2007; Hoyer et al., 2019; Kanazawa et al., 2022; Noguchi et al., 2021; Wang et al., 2024; Zhang et al., 2023). Given the feasibility limitations of dynamic monitoring in clinical practice, this study primarily focused on exploring the association between baseline inflammatory status and short-term functional outcomes.

### Statistic analysis

**FIGURE 2:**
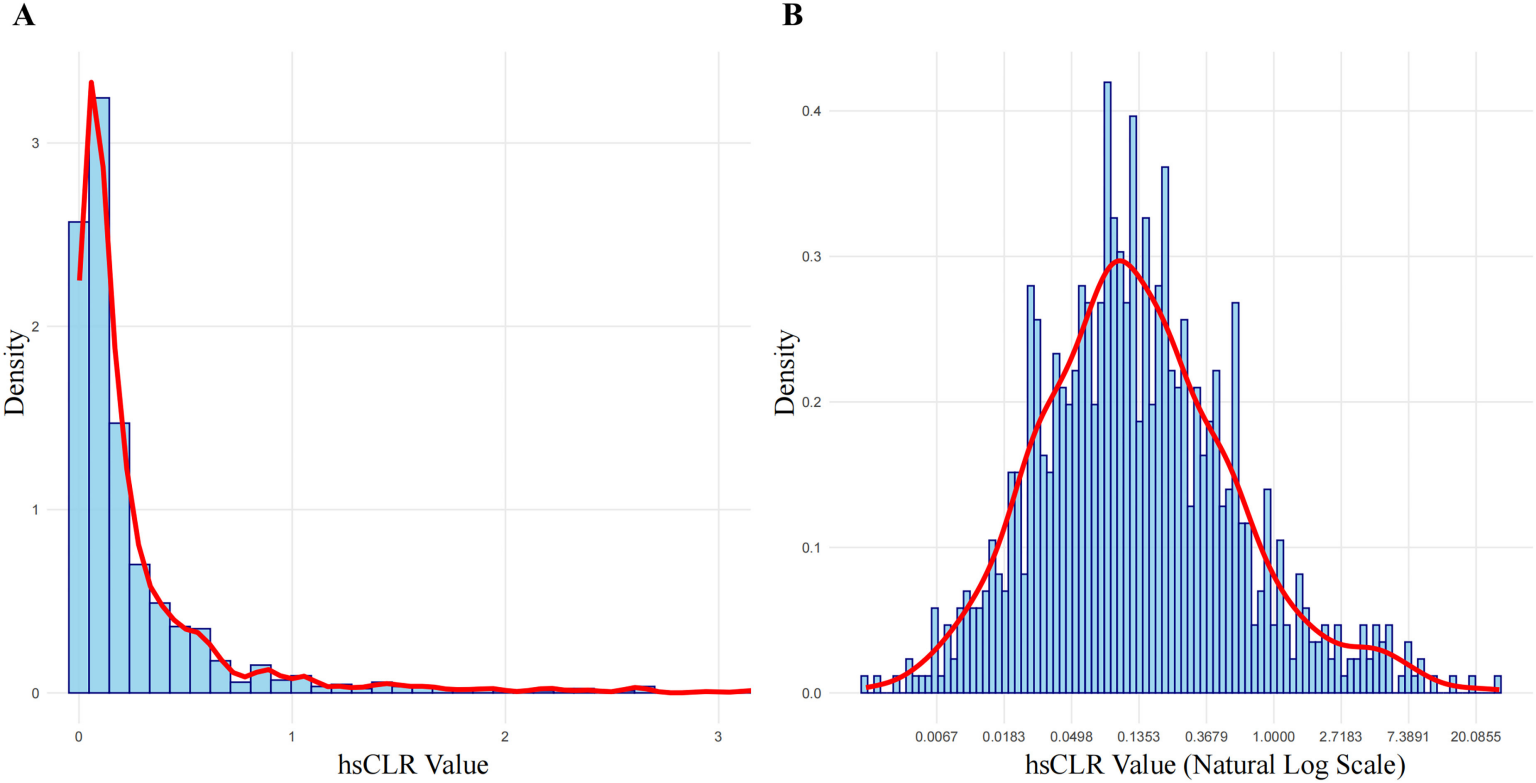
Comparative analysis of hsCLR distribution. **(A)** Linear Scale (hsCLR). **(B)** Natural Logarithm Scale (lnhsCLR).

Categorical variables were expressed as case counts (%). Group differences across hsCLR quartiles (Q1-Q4) were assessed using ANOVA for normally distributed continuous variables, Kruskal-Wallis tests for non-normally distributed continuous variables, and χ^2^ tests or Fisher’s exact tests for categorical variables, as appropriate. Given the skewed distribution of raw hsCLR values, natural logarithmic transformation (lnhsCLR) was performed to facilitate subsequent analyses. Figure 2 compares the distribution of the original hsCLR values and their logarithmically transformed values (lnhsCLR).

Multivariate logistic regression analyses were used to evaluate the association between the levels of lnhsCLR and short-term unfavorable functional outcome in AIS patients undergoing IVT, with the estimation of the odds ratio (OR) and 95% confidence interval (95% CI). In model 1, an unadjusted analysis was conducted. Model 2 was adjusted for age, gender, current smoking and current drinking alcohol. Model 3 added more adjustments, including history of DM, history of hypertension, history of AF, history of CHD, PAO, stroke subtype, OTT, antiplatelet therapy, anticoagulation therapy and NIHSS group. Candidate variables were determined according to prior clinical evidence, published studies in the field, and clinical relevance. Multicollinearity among independent variables was evaluated using the variance inflation factor (VIF) calculated from the multivariate logistic regression model. For categorical variables, adjusted VIF (Adj-VIF) was computed as GVIF^1/(2 × *Df*)^. Variables with Adj-VIF < 3 were considered free of severe multicollinearity (Supplementary Table 1).

We further employed restricted cubic spline (RCS) model, adjusted by Model 3, to analyze the dose-response relationship between the levels of lnhsCLR and short-term unfavorable functional outcome in AIS patients undergoing IVT. Moreover, we evaluated the predictive value of hsCLR using the receiver operating characteristic (ROC) curve and the area under the curve (AUC), and DeLong’s test was adopted for pairwise comparison of AUC values between different biomarkers. In addition, net reclassification index (NRI), and integrated discrimination improvement (IDI) were used to evaluate the incremental prognostic value of lnhsCLR beyond conventional risk factors. Statistical analysis was performed using R version 4.5.2 software.^1^ A value of *P* < 0.05 in the two-tailed test was considered to be statistically significant.

## Results

### Baseline characteristics of AIS patients treated with IVT

**FIGURE 3:**
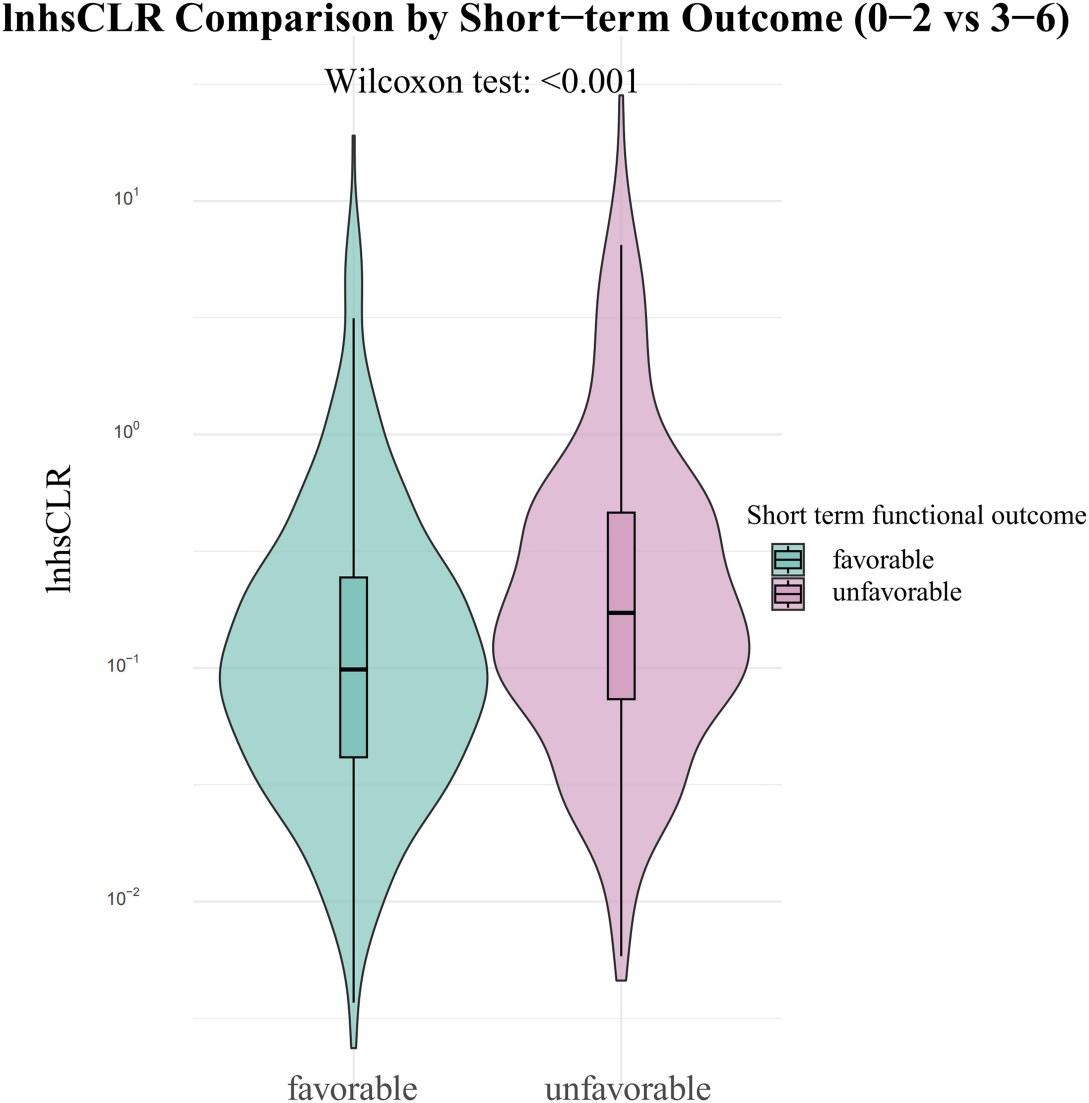
Comparison of lnhsCLR levels between patients with favorable and unfavorable short-term functional outcome.

**FIGURE 4:**
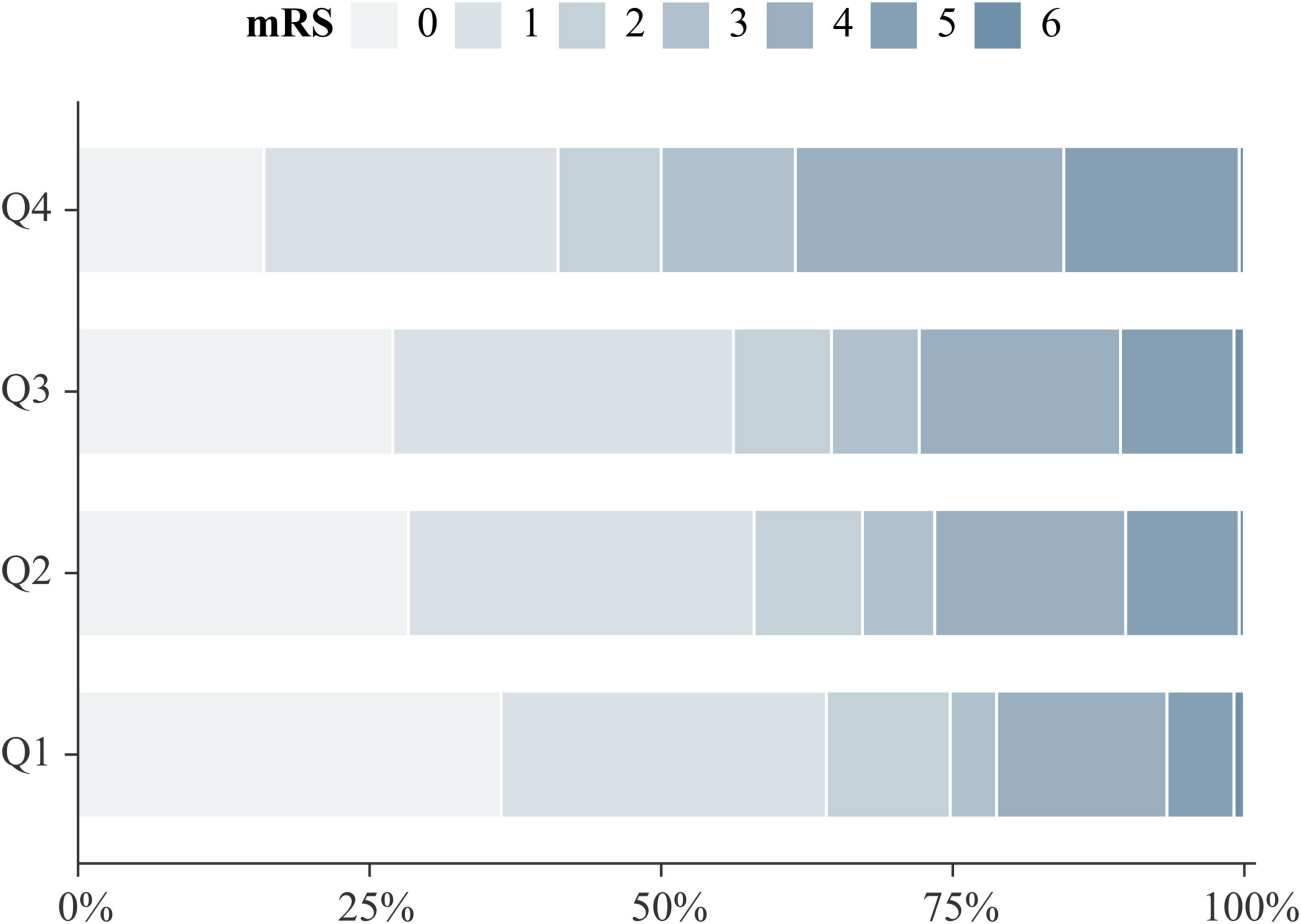
Distribution of discharge mRS scores among the four quartiles of hsCLR.

A total of 904 patients were enrolled in this study. Table 1 presented the baseline characteristics of all participants stratified by hsCLR quartiles. Notably, significant differences were observed in baseline characteristics across different hsCLR quartiles, including hypertension (*P* = 0.018), AF (*P* = 0.010), PAO (*P* < 0.001), NIHSS group (*P* = 0.004), stroke subtype (*P* = 0.038), OTT (*P* = 0.003), anticoagulation therapy (*P* = 0.037) and short-term functional outcome (*P* < 0.001). Figure 3 demonstrated that the levels of lnhsCLR in the unfavorable group were significantly higher than that in the favorable group (*P* < 0.05). Based on the ordered regression model in Figure 4, there was a significant correlation between the hsCLR quartile grouping (Q1–Q4) and the distribution of mRS scores at discharge (*P* for trend < 0.001), with the Q4 group showing significantly higher mRS scores than the Q1 group.

**TABLE 1:** Characteristics of participants based on the quartile of hsCLR.

Characteristics	hsCLR				*P*
	Q1 (*n* = 226)	Q2 (*n* = 226)	Q3 (*n* = 226)	Q4 (*n* = 226)	
Age, (%)					0.105
≥ 65	150 (66.37)	168 (74.34)	165 (73.01)	172 (76.11)	
<65	76 (33.63)	58 (25.66)	61 (26.99)	54 (23.89)	
Gender, (%)					0.096
Female	82 (36.28)	101 (44.69)	79 (34.96)	79 (34.96)	
Male	144 (63.72)	125 (55.31)	147 (65.04)	147 (65.04)	
Vascular risk factors, (%)					
Current drinking alcohol	67 (29.65)	62 (27.43)	70 (30.97)	69 (30.53)	0.848
Current smoking	65 (28.76)	78 (34.51)	70 (30.97)	71 (31.42)	0.622
History of diabetes mellitus	50 (22.12)	56 (24.78)	55 (24.34)	53 (23.45)	0.916
History of hypertension	139 (61.50)	142 (62.83)	151 (66.81)	168 (74.34)	0.018
History of atrial fibrillation	37 (16.37)	40 (17.70)	43 (19.03)	63 (27.88)	0.010
History of coronary heart disease	12 (5.31)	15 (6.64)	16 (7.08)	16 (7.08)	0.854
Clinical assessment, (%)					
PAO	85 (37.61)	88 (38.94)	104 (46.02)	130 (57.52)	< 0.001
NIHSS group					0.004
Mild (0–5)	119 (52.65)	113 (50.00)	106 (46.90)	83 (36.73)	
Severe ( ≥ 6)	107 (47.35)	113 (50.00)	120 (53.10)	143 (63.27)	
Stroke subtype (TOAST)					0.038
LAA	101 (44.69)	113 (50.00)	115 (50.88)	108 (47.79)	
CE	51 (22.57)	54 (23.89)	60 (26.55)	75 (33.19)	
SAO	53 (23.45)	47 (20.80)	36 (15.93)	33 (14.60)	
Other/unknown	21 (9.29)	12 (5.31)	15 (6.64)	10 (4.42)	
OTT					0.003
< 3 h	172 (76.11)	160 (70.80)	166 (73.45)	138 (61.06)	
3–4.5 h	54 (23.89)	66 (29.20)	60 (26.55)	88 (38.94)	
Medication use, (%)					
Antiplatelet therapy					0.107
Never use	24 (10.62)	37 (16.37)	33 (14.60)	46 (20.35)	
New initiation post-stroke	176 (77.88)	171 (75.66)	175 (77.43)	158 (69.91)	
Use prior to stroke	26 (11.50)	18 (7.96)	18 (7.96)	22 (9.73)	
Anticoagulation therapy					0.037
Never use	160 (70.80)	162 (71.68)	150 (66.37)	135 (59.73)	
New initiation post-stroke	57 (25.22)	48 (21.24)	66 (29.20)	78 (34.51)	
Use prior to stroke	9 (3.98)	16 (7.08)	10 (4.42)	13 (5.75)	
Short term functional outcome, (%)					< 0.001
Favorable	169 (74.78)	152 (67.26)	146 (64.60)	113 (50.00)	
Unfavorable	57 (25.22)	74 (32.74)	80 (35.40)	113 (50.00)	

hsCLR, high-sensitivity C-reactive protein/lymphocyte ratio; NIHSS, National Institutes of Health Stroke Scale; OTT, onset to treatment time; PAO, proximal arterial occlusion; TOAST, Trial of Org 10172 in Acute Stroke Treatment; LAA, large-artery atherosclerosis; CE, cardioembolism; SAO, small-artery occlusion; SOE, stroke of other determined etiology; SUE, stroke of undetermined etiology. Comparisons across hsCLR quartiles were performed using ANOVA, Kruskal-Wallis test, χ^2^ test, or Fisher’s exact test as appropriate.

### Relationship between lnhsCLR and short-term unfavorable functional outcome

**FIGURE 5:**
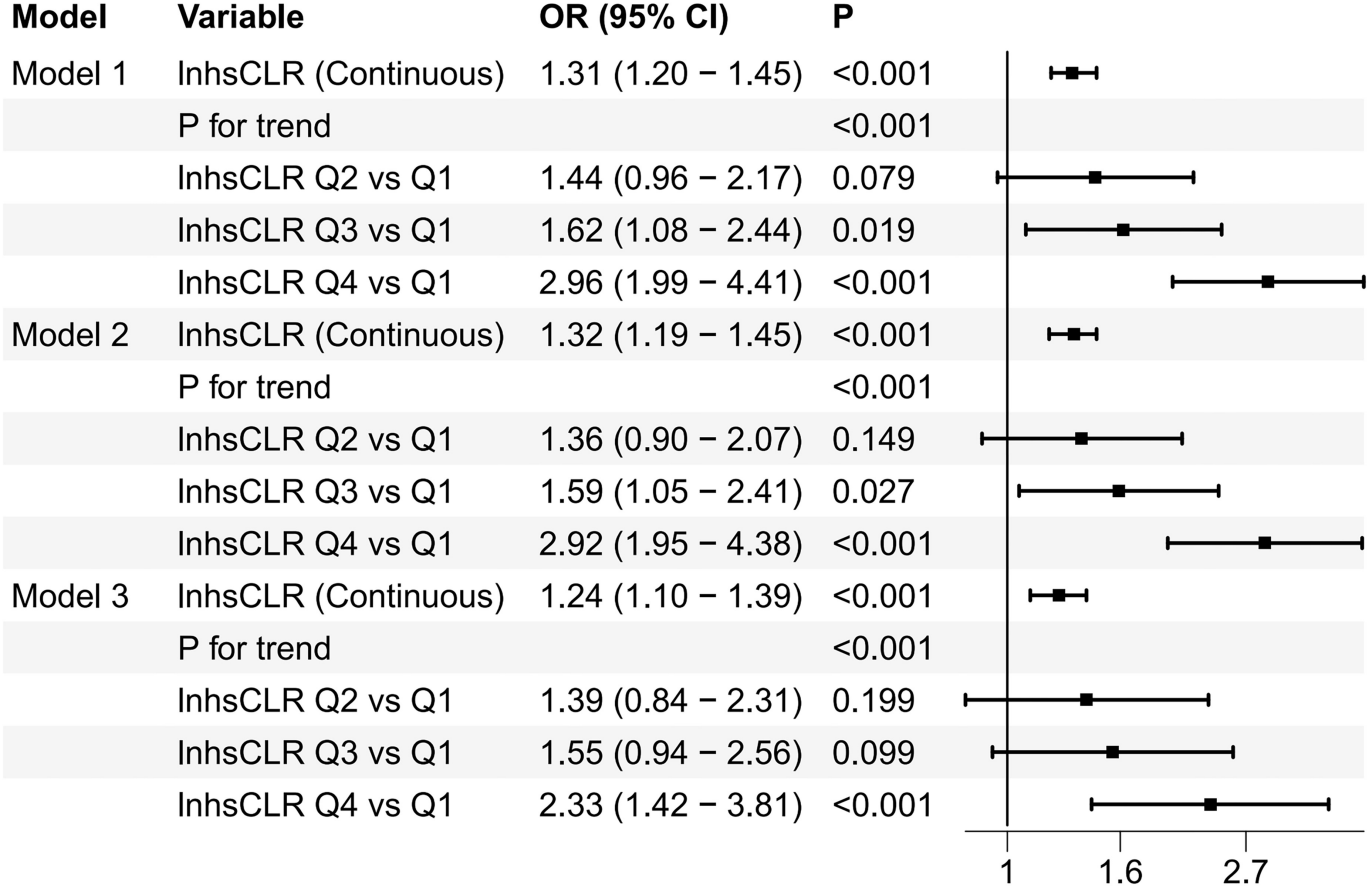
Odds ratios (95% confidence intervals) of lnhsCLR and post-thrombolysis short-term outcome in different models.

The results of multivariate logistic regression analysis between the elevated levels of lnhsCLR and short-term unfavorable functional outcome are presented in Figure 5. In the multivariate logistic regression model, elevated levels of lnhsCLR were positively associated with the risk of short-term unfavorable functional outcome. After adjusting for multiple confounding factors (model 3), each unit increase in lnhsCLR corresponded to a 24% higher risk of unfavorable outcome (OR = 1.24; 95% CI: 1.10–1.39; *P* < 0.001; Figure 5). Furthermore, the elevation of hsCLR quartiles (Q1–Q4) demonstrated a significant and consistent dose-response relationship (*P* for trend < 0.001). After adjustment by model 3, the highest group (Q4) exhibited a more than twofold increased risk of unfavorable functional outcome at discharge compared to the lowest group (Q1) (OR = 2.33; 95% CI: 1.42–3.81; *P* < 0.001; Figure 5).

### RCS analysis

**FIGURE 6:**
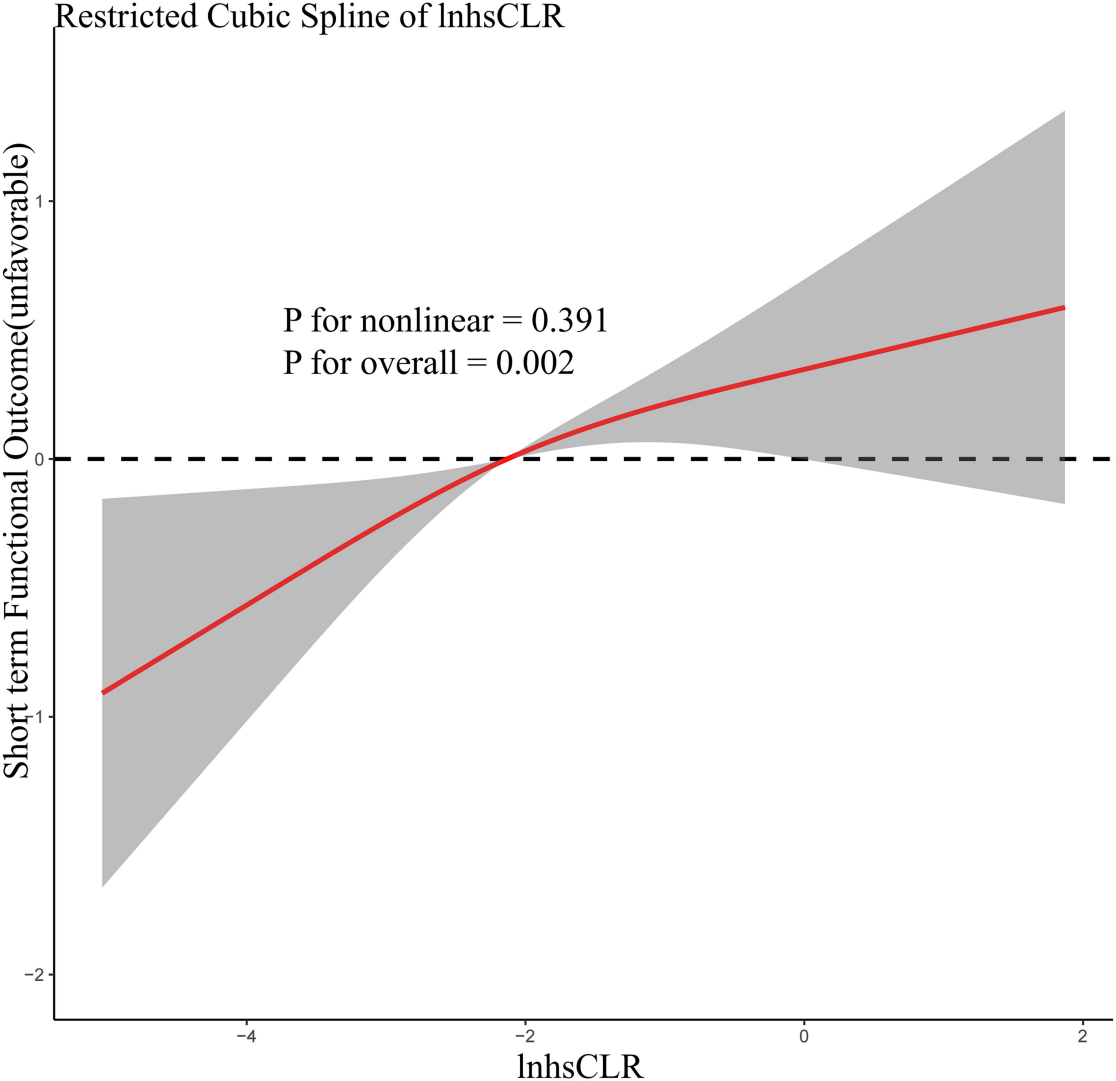
Restricted cubic spline analysis of lnhsCLR and unfavorable post-thrombolysis short-term outcome.

Figure 6 presented the RCS analysis result of the dose-response relationship between the levels of lnhsCLR and short-term unfavorable functional outcome, revealing a significant positive linear association (*P* for overall = 0.002; *P* for nonlinear = 0.391).

### ROC curve analysis

**FIGURE 7:**
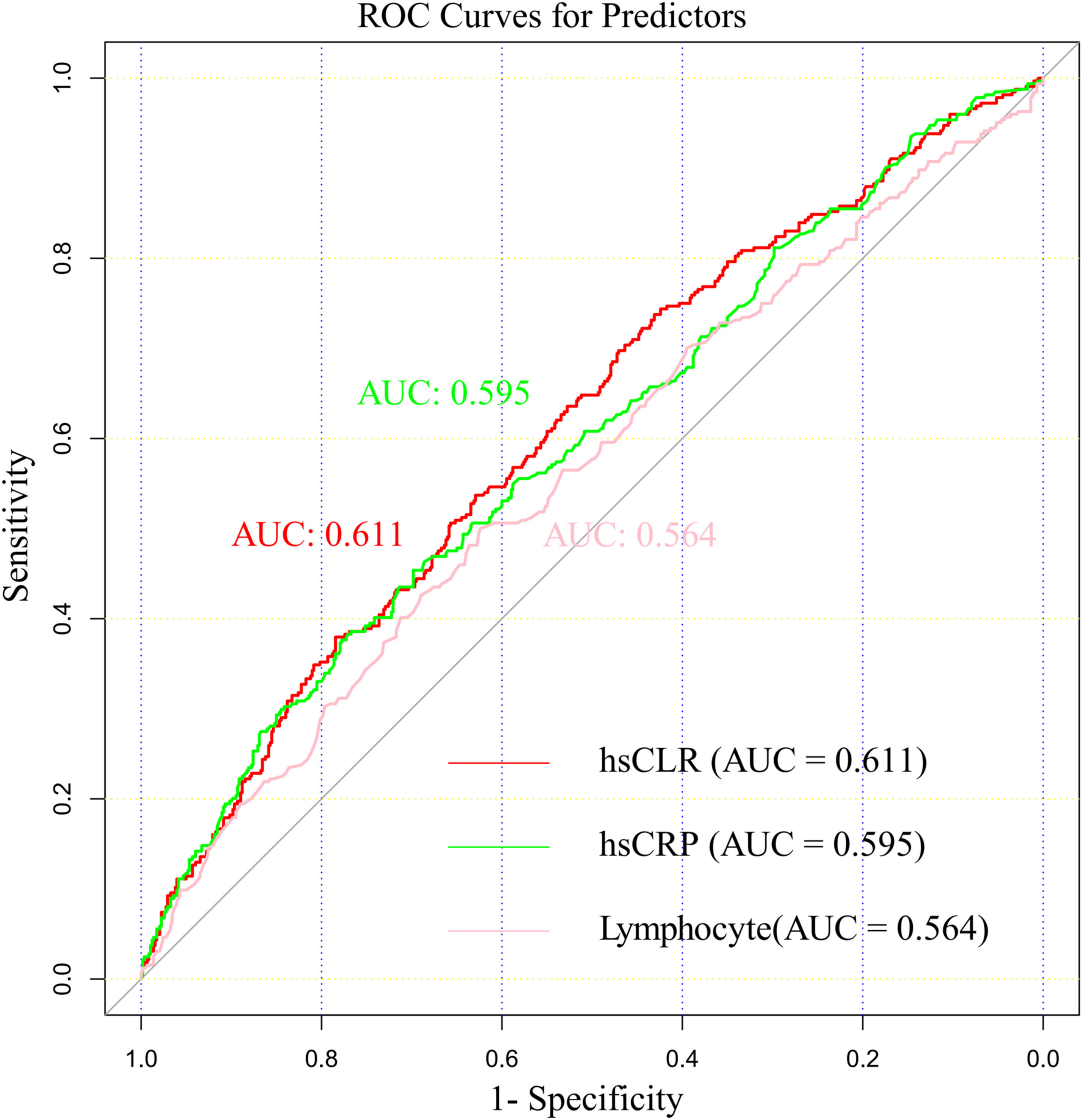
Predictive value of hsCLR, hsCRP and lymphocyte for unfavorable post-thrombolysis short-term outcome.

Figure 7 compared the predictive efficacy of hsCLR, hsCRP, and lymphocyte count for unfavorable short-term functional outcome. The corresponding AUC values (95% CI) were 0.611 (0.573–0.650) for hsCLR, 0.595 (0.556–0.633) for hsCRP, and 0.564 (0.525–0.604) for lymphocyte. The predictive value of hsCLR was statistically superior to that of high-sensitivity C-reactive protein (AUC: 0.611 vs. 0.595, DeLong’s test, *Z* = 2.22, *P* = 0.026) and lymphocyte count (AUC: 0.611 vs. 0.564, DeLong’s test, *Z* = 2.19, *P* = 0.028), though its overall discriminatory performance was modest and requires further validation to confirm clinical applicability.

### NRI and IDI analysis

As shown in Table 2, the addition of lnhsCLR to the conventional risk model significantly improved the prediction of unfavorable short-term functional outcome (mRS score 3–6) in AIS patients. NRI indicated a robust enhancement of 21.22% (*P* = 0.002). Furthermore, the IDI increased by 1.21% (*P* = 0.002), presenting incremental discriminative ability of lnhsCLR.

**TABLE 2:** Discrimination and reclassification statistics for short-term unfavorable functional outcome by lnhsCLR among patients with acute ischemic stroke.

Characteristic	NRI (continuous), %	IDI, %
Short-term unfavorable functional outcome	Estimate (95 % CI), *P-*value	Estimate (95 % CI), *P*-value
Conventional model	Reference	Reference
Conventional model + lnhsCLR	21.22 (7.30–34.32), 0.002	1.21 (0.04–2.00), 0.002

NRI, net reclassification improvement; IDI, integrated discrimination index. Conventional model included age, gender, current smoking, current drinking alcohol, history of diabetes mellitus, history of hypertension, history of atrial fibrillation, history of coronary heart disease, proximal arterial occlusion, TOAST group, onset to treatment time, antiplatelet therapy, anticoagulation therapy and NIHSS group.

## Discussion

Based on current evidence, the present study is the first to investigate the association between hsCLR and short-term unfavorable functional outcomes in AIS patients undergoing intravenous thrombolysis, with a relatively large sample size. As shown in Table 1, several key clinical confounders, including hypertension, atrial fibrillation, PAO, baseline NIHSS score, TOAST subtype, OTT, and anticoagulant use, differed significantly across hsCLR quartiles. Elevated hsCLR was associated with a higher burden of vascular risk factors and more severe stroke severity, likely reflecting intensified systemic inflammation and immune dysregulation induced by chronic cardiovascular diseases and acute ischemic brain injury (Li et al., 2018; Su et al., 2020). Variations in hsCLR across TOAST subtypes may also correspond to differences in inflammatory activity related to distinct stroke etiologies (Lehmann et al., 2015; Meisinger et al., 2024). To minimize confounding, these variables were adjusted for in the fully adjusted logistic regression model (Model 3). Our results demonstrated that higher baseline hsCLR levels were independently associated with an increased risk of unfavorable short-term functional outcome at discharge. Furthermore, RCS analysis revealed a linear dose-response relationship between lnhsCLR and poor functional outcome. These findings collectively suggested that hsCLR may be a risk factor for short-term unfavorable functional outcome in AIS patients treated with IVT.

Previous studies have consistently shown that inflammation plays a key role in the occurrence and progression of stroke, and is crucial for prognosis. In recent years, composite inflammatory parameters derived from complete blood count and blood biochemical indicators have attracted significant attention in stroke research. Studies demonstrated that hsCRP may be an effective predictor of poor prognosis in stroke patients (Jiang et al., 2021; Xu et al., 2022). In parallel, lymphocyte also play a significant role in stroke prognosis (Wu et al., 2023). Recently, hsCLR has been proposed as a novel biomarker for systemic inflammation and immune response. According to previous study, hsCLR were related to a heightened risk of COPD, with nonlinear patterns and threshold effects observed (Ao et al., 2025). One research showed a link between pre-surgery hsCLR levels and the likelihood of incision complications after medial opening-wedge high tibial osteotomy for varus knee osteoarthritis (Ji et al., 2025). What is more, hsCLR at admission was reported to be independently related to post-mechanical thrombectomy adverse outcomes in AIS patients (Dogan et al., 2025). While these studies established hsCLR as an effective inflammatory biomarker among several diseases, its role in the distinct pathophysiological context of IVT—which involves reperfusion injury and a potentially different inflammatory cascade compared to mechanical thrombectomy—remained unexplored. Our study, with a relatively large sample size, indicated that there may be a significant positive linear association between the levels of lnhsCLR and short-term unfavorable functional outcome at discharge, which could further support the importance of hsCLR in clinical prognosis research of AIS. In addition, the results of our studies also showed that hsCLR could have a greater predictive value in comparison with either hsCRP or lymphocyte count alone, implying that hsCLR, integrating the advantages of acute-phase response and adaptive immunity, may more comprehensively capture post-thrombolysis complex inflammatory state and forecasting disease prognosis.

Nonetheless, it is important to admit that our study has a few drawbacks that need to be identified and addressed in future investigations. First, this study was conducted only in China, and the lack of an external validation cohort may limit the generalizability of our findings to other ethnic and geographic populations. Future multi-center, prospective studies with external validation are warranted to verify the broader clinical applicability of our results. Second, we only collected baseline levels of hsCRP and lymphocyte upon admission. The lack of serial measurements prevents us from assessing the dynamic changes of these parameters during hospitalization. In our future research, we will add dynamic monitoring of inflammatory biomarkers at multiple time points, which can more accurately reflect the dynamic evolution of systemic inflammation and immune status, thereby further improving the understanding and interpretation of the relationship between inflammatory responses and patient outcomes. Furthermore, the outcome assessment in this study was limited to short-term functional status at discharge only, without longer-term follow-up data. This relatively brief follow-up period restricts our ability to explore the associations of these biomarkers with long-term functional recovery, mortality, or stroke recurrence beyond the acute phase. Future studies with extended and standardized follow-up durations are warranted to address this issue. Third, another recognized limitation is the incomplete ascertainment of all relevant risk factors of ischemic stroke. This could result in confounding bias. Future prospective studies designed to comprehensively capture a broader panel of genetic, environmental, and behavioral determinants are needed to validate and refine our risk estimates. In addition, the predictive accuracy of hsCLR alone was modest, with an AUC of 0.611, suggesting its relatively limited standalone clinical utility. However, further NRI and IDI analyses revealed that adding hsCLR to conventional clinical prognostic models significantly enhanced their predictive capacity, beyond that achieved with established clinical variables alone. Accordingly, hsCLR may act as a useful inflammatory biomarker. Several inflammatory biomarkers such as Complement C3, S100A8/A9, the white blood cell to high-density lipoprotein cholesterol ratio have been widely recognized as prognostic indicators in acute ischemic stroke (Guo et al., 2020; Wang et al., 2025; Yang et al., 2021). Future investigations are warranted to directly compare the prognostic performance of hsCLR with these established biomarkers to further clarify its clinical value. However, the predictive value of hsCLR was not directly compared with well-established clinical prognostic tools such as the NIHSS score, which should be recognized as a limitation of this study.

Despite these limitations, the present study provides valuable preliminary evidence. To the best of our knowledge, this is the first study to identify hsCLR—a routine, easily measured and inexpensive biomarker—as a potential predictor of short-term prognosis in AIS patients undergoing intravenous thrombolysis. Our findings may thus provide a novel candidate indicator for prognostic stratification. Although further validation is warranted, the present results may shed light on future biomarker research and improve our understanding of the inflammatory mechanisms underlying early neurological recovery in AIS patients treated with IVT.

## Data Availability

The raw data supporting the conclusions of this article will be made available by the authors, without undue reservation.

## Footnotes

1
http://www.R-project.org/
