# Supplementary material for: Relationship between high-sensitivity C-reactive protein/lymphocyte ratio and post-thrombolysis short-term outcome in acute ischemic stroke patients
Source: Front Aging Neurosci. 2026 May 29;18:1839899. doi: 10.3389/fnagi.2026.1839899 (PMC13260141; doi:10.3389/fnagi.2026.1839899)
Supplement: Supplementary file 1 [file Table_1.docx]

| **Variable** | **Adj-VIF** |
| --- | --- |
| Atrial fibrillation | 1.42 |
| Current smoking | 1.35 |
| Current drinking alcohol | 1.30 |
| Anticoagulation therapy | 1.22 |
| Gender | 1.20 |
| Stroke subtype (TOAST) | 1.19 |
| PAO | 1.07 |
| Age | 1.07 |
| Antiplatelet therapy | 1.06 |
| History of coronary heart disease | 1.06 |
| History of diabetes mellitus | 1.05 |
| NIHSS group | 1.03 |
| History of hypertension | 1.03 |
| lnCLR | 1.02 |
| OTT | 1.02 |

**Supplementary Table 1. Assessment of multicollinearity for all variables.**
